# In Vivo Evaluation of Dual-Targeted Nanoparticles Encapsulating Paclitaxel and Everolimus

**DOI:** 10.3390/cancers11060752

**Published:** 2019-05-29

**Authors:** Loujin Houdaihed, James Christopher Evans, Christine Allen

**Affiliations:** Leslie Dan Faculty of Pharmacy, University of Toronto, Toronto, ON M5S 3M2, Canada; loujin.houdaihed@mail.utoronto.ca (L.H.); james.evans@utoronto.ca (J.C.E.)

**Keywords:** nanoparticles, drug combination, paclitaxel, everolimus, dual-targeting, breast cancer

## Abstract

A synergistic combination of paclitaxel (PTX) and everolimus (EVER) can allow for lower drug doses, reducing the toxicities associated with PTX, while maintaining therapeutic efficacy. Polymeric nanoparticles (NPs) of high stability provide opportunities to modify the toxicity profile of the drugs by ensuring their delivery to the tumor site at the synergistic ratio while limiting systemic drug exposure and the toxicities that result. The goal of the current study is to evaluate the in vivo fate of human epidermal factor receptor 2 (HER2) and epidermal growth factor receptor (EGFR) dual-targeted PTX+EVER-loaded NPs (Dual-NPs) in an MDA-MB-231-H2N breast cancer (BC) tumor-bearing mouse model. The pharmacokinetic parameters, plasma area under the curve (AUC) and half-life (t_1/2_z) were found to be 20-fold and 3 to 4-fold higher, respectively, for the drugs when administered in the Dual-NPs in comparison to the free-drug combination (i.e., PTX+EVER) at an equivalent dose of PTX. While maintaining anti-tumor efficacy, the levels of body weight loss were significantly lower (*p* < 0.0001) and the overall degree of neurotoxicity was reduced with Dual-NPs treatment in comparison to the free-drug combination when administered at an equivalent dose of PTX. This study suggests that Dual-NPs present a promising platform for the delivery of the PTX and EVER combination with the potential to reduce severe PTX-induced toxicities and in turn, improve quality of life for patients with BC.

## 1. Introduction

Paclitaxel (PTX)-induced toxicities continue to be one of the main challenges associated with the use of this agent. PTX has several dose-limiting adverse effects including neutropenia and neurotoxicity. Additionally, the commercial formulation of PTX (Taxol) has been associated with severe hypersensitivity reactions, mainly caused by the excipients used for drug solubilization (i.e., ethanol and Cremophor EL) [1].

Over the years, drug-combinations have emerged as one promising avenue for the treatment of cancer. We can take advantage of the synergistic effects of drug-combinations with the administration of doses that are much lower than the maximum tolerated doses (MTDs) of the drugs. This can lead to a reduction in systemic toxicity, while improving or at least maintaining the desired therapeutic effect [2,3,4,5].

The combination of the chemotherapeutic agent PTX, and the mammalian target of rapamycin (mTOR) inhibitor everolimus (EVER) has previously been shown to have pronounced synergistic effects in breast cancer (BC) cells in vitro [6]. Despite the potential of this drug combination, only modest efficacy was seen in clinical studies conducted to date—with no significant improvements with respect to toxicity (e.g., NCT00876395 and NCT00915603) [7,8]. This outcome may be attributed to the distinct pharmacokinetic profiles of the drugs, and the administration of agents at their MTDs or standard doses, thereby preventing the delivery of the drugs to the tumor at the optimal synergistic ratio, which hampers the potential synergistic effects.

Recent advances in cancer nanomedicines provide unique opportunities to modify the toxicity profile of the drugs by increasing their delivery to the target site while limiting systemic drug exposure and the toxicities that result. Vyxeos^®^ (Jazz Pharmaceuticals, Dublin, Ireland) is a promising “two-in-one” formulation that includes liposomes that co-encapsulate the combination of cytarabine and daunorubicin at a synergistic molar ratio of 5:1. Vyxeos was approved in 2017 by the FDA for treating patients with high risk acute myeloid leukemia (AML) [9]. In a Phase III clinical trial (NCT01696084), Vyxeos demonstrated good tolerability and significant improvements in the overall survival of patients compared to the free-drug combination in patients with AML [10].

**Scheme 1:**
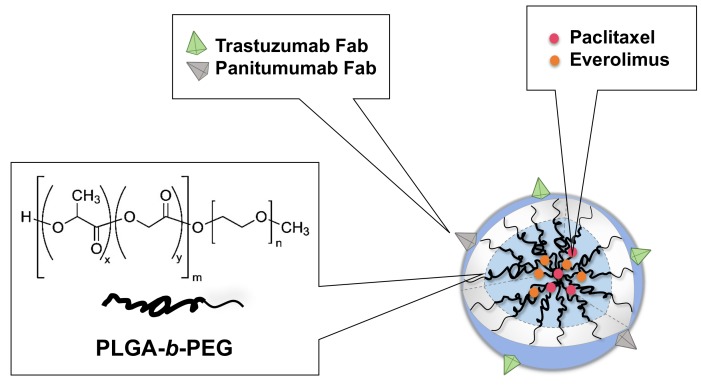
Graphical schematic depicting the composition of dual-targeted paclitaxel (PTX)+everolimus (EVER)-loaded nanoparticles (Dual-NPs). Adapted with permission from “Loujin Houdaihed, James C. Evans, Christine Allen. Codelivery of Paclitaxel and Everolimus at the Optimal Synergistic Ratio: A Promising Solution for the Treatment of Breast Cancer. *Molecular Pharmaceutics*. **2018**, 15(9): 3672-3681”. Copyright © (2018), American Chemical Society.

We have developed and characterized PTX+EVER-loaded NPs composed of poly (ethylene glycol)-*b*-poly(lactide-co-glycolide) copolymer (mPEG_5000_-*b*-PLGA_15,800_, 50:50 LA/GA). The NPs are actively targeted to human epidermal factor receptor 2 (HER2) and epidermal growth factor receptor (EGFR), using HER2-targeted trastuzumab (TmAb) and EGFR-targeted Panitumumab (PmAb) Fab fragments, to deliver the drug combination to BC cells (Scheme 1, Table 1) [11].

Active targeting has been widely utilized to increase the cellular internalization of anticancer therapies into cancer cells, with a goal towards improving therapeutic effects [12]. HER2 is overexpressed in 15–25% of breast cancer (BC) [13], whereas EGFR is found in 15–20% of BC and in up to 60% of basal triple-negative BC. Importantly, up to 35% of HER2+ BC co-expresses EGFR [14]. In addition, patients with tumors co-expressing both receptors demonstrate the shortest survival times when compared to those with tumors positive for only one of the receptors [14]. Taken together, HER2 and EGFR represent two attractive complementary targets for the design of a therapy against HER2+/EGFR+ BC.

The current study builds on the promising results seen with Dual-NPs in vitro and examines the pharmacokinetics and biodistribution profiles of PTX and EVER when administered in the Dual-NPs compared with the free-drug combination in a relevant animal model of BC. Furthermore, the therapeutic effect of PTX and EVER administered in Dual-NPs was evaluated in mice bearing MDA-MB-231-H2N BC tumors which co-express HER2 and EGFR (HER2_mod_/EGFR_mod_). Additionally, the safety profile of Dual-NPs relative to the free-drug combination was evaluated by examining neurotoxicity and hemotoxicity post administration. This study demonstrates that Dual-NPs have high stability in vivo and, subsequently, result in significant improvements with regard to safety compared to the free-drug combination in the MDA-MB-231-H2N BC tumor-bearing mouse model.

## 2. Results

### 2.1. Preparation and Characterization of Dual-NPs

PTX and EVER were co-encapsulated at the optimal synergistic ratio within poly(ethylene glycol)-*b*-poly(lactide-*co*-glycolide) (mPEG_5000_-*b*-PLGA_15,800_, 50:50 LA/GA) polymeric nanoparticles. TmAb(Fab) and PmAb(Fab) were prepared, purified and conjugated to the surface of PTX+EVER-loaded NPs via amine coupling. The physicochemical characteristics of the Dual-NP are summarized in Table 1.

### 2.2. Pharmacokinetic Study

**Figure 1:**
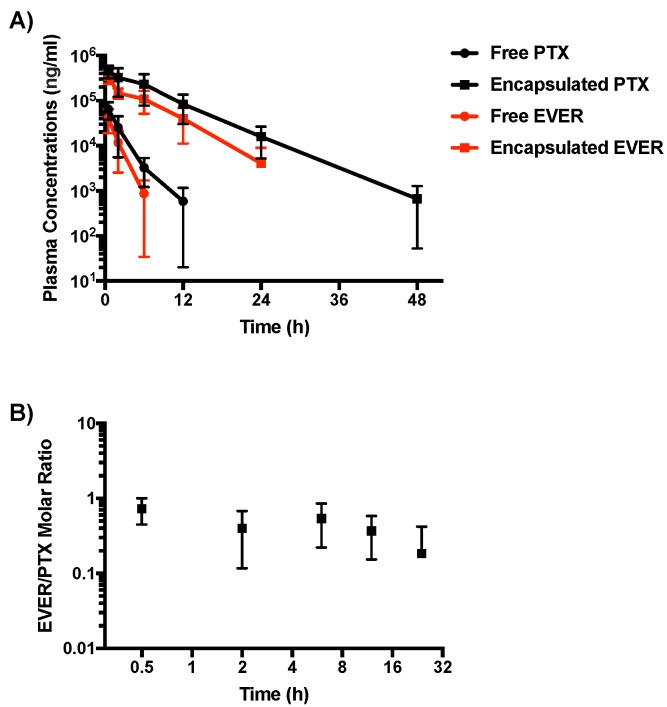
(**A**) Plasma concentrations of paclitaxel (PTX) and everolimus (EVER) after single intravenous administration of dual-targeted PTX+EVER-loaded nanoparticles (Dual-NPs) or free PTX+EVER combination to healthy BALB/c female mice at a PTX-equivalent dose of 15 mg/kg and EVER-equivalent dose of 7.5 mg/kg. (**B**) EVER:PTX molar ratio of drugs released from Dual-NPs in the plasma. (*n* = 5).

Healthy BALB/c female mice were given a single intravenous injection of either the free PTX+EVER combination or Dual-NPs at a PTX-equivalent dose of 15 mg/kg and EVER-equivalent dose of 7.5 mg/kg (at a molar ratio of 1:0.5). The levels of PTX and EVER were measured in the plasma at various timepoints (Figure 1A) and the pharmacokinetic parameters of each group were calculated using non-compartmental analysis (Table 2). Both, PTX and EVER encapsulated in Dual-NPs exhibited slower clearance from the plasma compared to the free-drug combination. While PTX and EVER in Dual-NPs were detectable in the plasma for up to 48 h and 24 h post-administration, respectively, free PTX and EVER were last detected in the plasma 12 h and 6 h post-administration, respectively. The area under the curve (AUC) for both PTX and EVER in Dual-NPs was approximately 20-fold higher than that of the free drug. The maximum plasma concentrations (C_max_) of PTX and EVER were 7-fold and 9-fold higher for Dual-NPs than for the free drugs, respectively. Furthermore, the half-life (t_1/2_z) was approximately 3 and 4 times greater for PTX and EVER, respectively, in Dual-NPs than for the free drugs. Analysis of the PTX:EVER molar ratio in the plasma revealed that Dual-NPs maintained the ratio of 1:0.5 for 24 h after administration (Figure 1B). This molar ratio had been found to be synergistic in in vitro studies conducted by our group [6]. The total drug concentrations found in plasma for mice administered the Dual-NPs reflect NP encapsulated and released drug. However, the rapid elimination of the free drugs from the circulation indicates that the majority of the PTX and EVER measured in the plasma are likely encapsulated within the NPs.

### 2.3. Biodistribution Study

**Figure 2:**
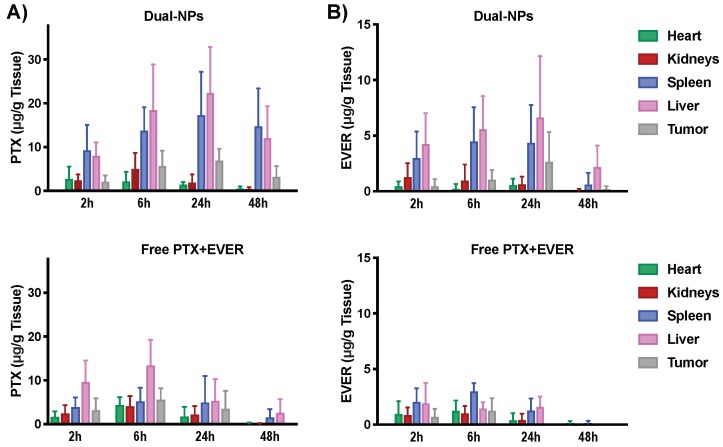
Tissue distribution of (**A**) paclitaxel (PTX) and (**B**) everolimus (EVER) in female Nonobese diabetic/severe combined immunodeficiency (NOD/SCID) mice bearing MDA-MB-231-H2N BC xenografts (HER2_mod_/EGFR_mod_) after single intravenous administration of dual-targeted PTX+EVER-loaded nanoparticles (Dual-NPs) or free PTX+EVER combination at a PTX-equivalent dose of 15 mg/kg and EVER-equivalent dose of 7.5 mg/kg. (*n* = 5).

Evaluation of the biodistribution of PTX+EVER (as a free-drug combination versus encapsulated within Dual-NPs) in tumor-bearing mice was performed at 2, 6, 24 and 48 h after administration. At 24 h post-administration, the Dual-NPs group showed a 2-fold increase in the tumor PTX accumulation compared to the levels detected in the free PTX+EVER group (*p* = 0.27, Figure 2). At 48 h post-administration, PTX was still detected in the tumor in the Dual-NPs group, however, PTX levels were below the limit of detection in the tumor in the free PTX+EVER group.

EVER accumulated in the tumor in the Dual-NPs group in a time dependent manner, achieving peak concentrations at 24 h, with values decreasing by 48 h. For EVER in the free PTX+EVER group, the drug levels in the tumor were below the limit of detection at 24 h and 48 h after administration. Importantly, the molar ratio of PTX:EVER that accumulated in the tumor in the Dual-NPs group at 24 h was 1:0.38 which remains close to the optimal synergistic ratio (1:0.5) found in vitro [6]. On the other hand, the optimal synergistic ratio of PTX:EVER was not seen in the tumor for the free PTX+EVER group at any time point. Overall, the levels of PTX and EVER seen in the tumors in mice treated with the Dual-NPs were higher relative to the levels seen for the free PTX+EVER group at 24 h and 48 h. However, at the earlier timepoints (2 and 6 h) the drug concentrations were comparable between the two groups.

Comparable levels of PTX and EVER were found to accumulate in the hearts and kidneys of mice in the Dual-NPs and free PTX+EVER groups. Differential accumulation of PTX and EVER following administration in Dual-NPs and free PTX+EVER occurred mainly in the spleen and liver. A 2–5 fold and 2–7 fold increase in liver and spleen uptake of PTX was seen in the Dual-NPs group relative to the free-drug combination group, while a 2–4 fold and 2–6 fold increase was noted in liver and spleen uptake of EVER, respectively, for the Dual-NPs group compared with the free-drug combination group. This outcome can be attributed to the clearance of the NPs via the mononuclear phagocyte system (MPS), which is one of the main elimination pathways for NPs [15].

### 2.4. Efficacy Studies

**Figure 3:**
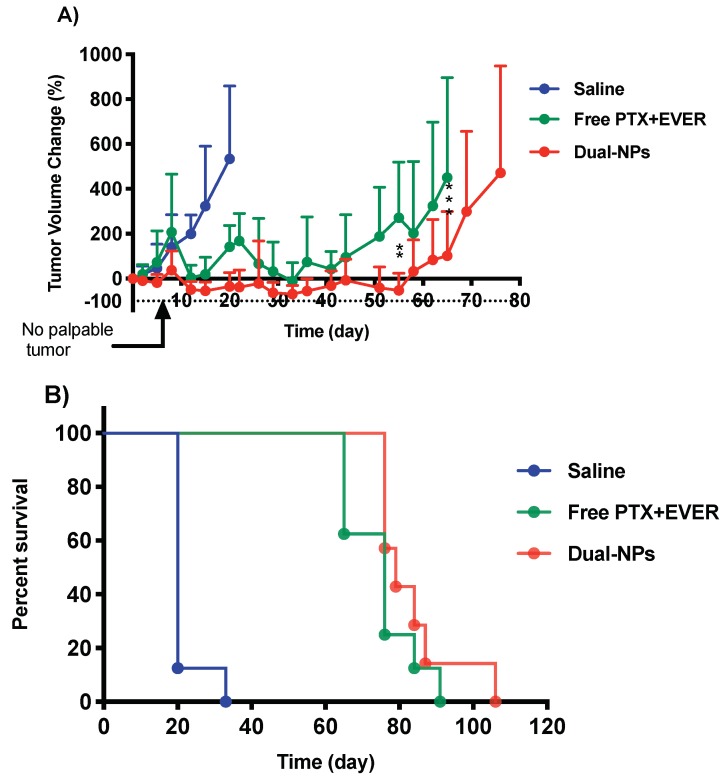
(**A**) Tumor growth inhibition of saline (blue), free paclitaxel and everolimus (PTX+EVER) (green), and dual-targeted PTX+EVER-loaded nanoparticles (Dual-NPs) (red) in female NOD/SCID mice bearing MDA-MB-231-H2N BC xenografts (HER2_mod_/EGFR_mod_) after intravenous administration of treatments at a PTX-equivalent dose of 15 mg/kg and EVER-equivalent dose of 7.5 mg/kg once a week for eight consecutive weeks. Data represent mean ± SD (*n* = 8). Two-way analysis of variance (ANOVA) was conducted where ** and *** indicate *p* = 0.001 and *p* = 0.0003, respectively. (**B**) Kaplan-Meier survival analysis.

**Figure 4:**
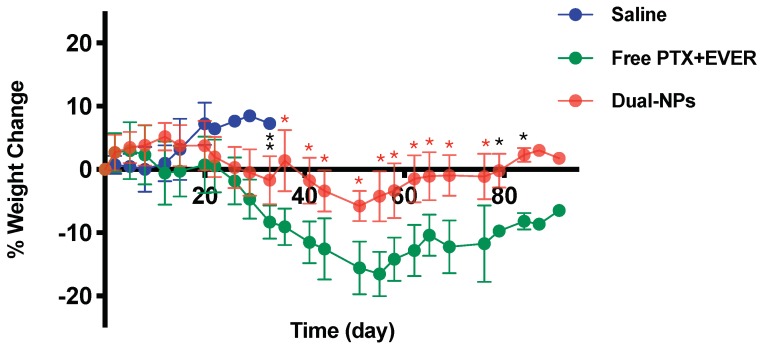
Animal body weight change in mice receiving saline (blue), free paclitaxel and everolimus (PTX+EVER) (green), and dual-targeted PTX+EVER-loaded nanoparticles (Dual-NPs) (red) from the first day of treatment to the ethical endpoint. Data represent mean ± SD (*n* = 8). Two-way analysis of variance (ANOVA) was conducted where *, **, and * indicate *p* < 0.04, *p* = 0.004, and *p* < 0.0001, respectively.

Tumor growth in NOD/SCID mice bearing subcutaneous (s.c.) MDA-MB-231-H2N BC tumors was inhibited following the administration of free PTX+EVER and Dual-NPs. Dual-NPs were associated with enhanced antitumor activity in comparison with the free-drug combination. The extent of tumor growth inhibition was significantly different at day 55 (*p* = 0.001) and day 65 (*p* = 0.0003) post initiation of treatment in the Dual-NPs group relative to the free-drug combination group (Figure 3A). In addition, the degree of body weight loss was significantly reduced in mice treated with the Dual-NPs compared with those receiving the same dose of the free PTX+EVER combination (*p* < 0.0001) (Figure 4). As shown in Figure 3B, Kaplan-Meier survival analysis revealed that the Dual-NPs resulted in comparable overall survival to that obtained with the free-drug combination. Median survival was 79 days for mice receiving Dual-NPs, and 76 days for those receiving the free PTX+EVER.

### 2.5. Toxicity Studies

**Figure 5:**
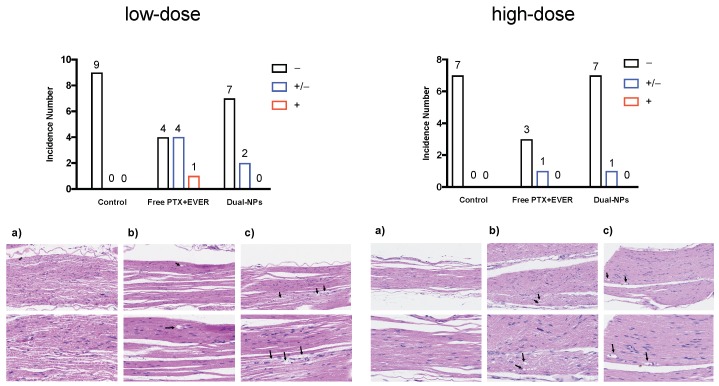
Degenerating myelinated nerve fibers (arrow) were examined in the sciatic nerve after staining with haematoxylin and eosin (H & E) two to three days after weekly injections for six weeks with saline (**a**), dual-targeted PTX+EVER-loaded nanoparticles (Dual-NPs) (**b**), and Free paclitaxel and everolimus (PTX+EVER) (**c**) at a PTX-equivalent dose of 8 mg/kg (left panel) or 15 mg/kg (right panel). Magnification, 20X (upper) and 40X (lower). A semi-quantitative scoring system was used in the evaluation of histologic sections of the sciatic nerve blinded as to the group. The scoring system addresses degenerative changes to myelinated nerve fibers (axonal swelling and vesicular degeneration). Several histologic sections, cut in a longitudinal plane, were examined from each sample. As previously described [16], the degenerating myelinated fiber score was defined as follows: –, no degenerative changes; +/−, very slight degree of degenerative changes (scattered, single fibers affected); +, slight degree of degenerative changes (scattered small groups of degenerative myelinated fibers).

Neurotoxicity was evaluated using histological examination of the sciatic nerve sections removed two to three days after the sixth weekly injection of saline, Dual-NPs, or free PTX+EVER (i.e., at the low dose of PTX (8 mg/kg, molar ratio of PTX:EVER of 1:0.5) (Figure 5, left panel). Mice in the Dual-NPs treatment group were found to have fewer degenerative myelinated fibers in contrast to mice treated with the free-drug combination. Only very slight degenerative changes were seen in sections of the sciatic nerve from two of nine mice in the Dual-NPs group. On the other hand, in the free-drug combination group, four out of nine mice showed very slight degenerative changes and one mouse showed slight degenerative changes in their sciatic nerve sections. Degenerative changes consisted of vesicular degeneration, while axonal swelling was not identified in any of the examined nerve sections.

The same study was repeated using a higher dose of PTX (15 mg/kg, molar ratio of PTX:EVER of 1:0.5) (Figure 5, right panel). Most sections of the sciatic nerve from mice in the Dual-NPs and free-drug combination groups were histologically unremarkable. Very slight degenerative changes, characterized by vesicular degeneration in a single nerve fiber or very few scattered individual fibers, were seen in the sections of the sciatic nerve from one mouse in the Dual-NPs group and one mouse in the free PTX+EVER group. Due to technical difficulties and incidence of death in the free PTX+EVER group early in the study, the number of sciatic nerve sections tested in the free PTX+EVER group was less than those in the Dual-NPs group, which makes further interpretation of the results difficult.

Blood samples from mice receiving the high-dose of PTX were evaluated for hemotoxicity and a complete blood count (CBC) analysis was performed using VetScan HM5 v2.2 (Abaxis, Union City, CA, USA). Considering the marked increase in Dual-NPs accumulation in the liver and spleen relative to free PTX+EVER, blood samples were also analyzed for biochemistry markers looking mainly for cues of liver and spleen toxicity.

**Figure 6:**
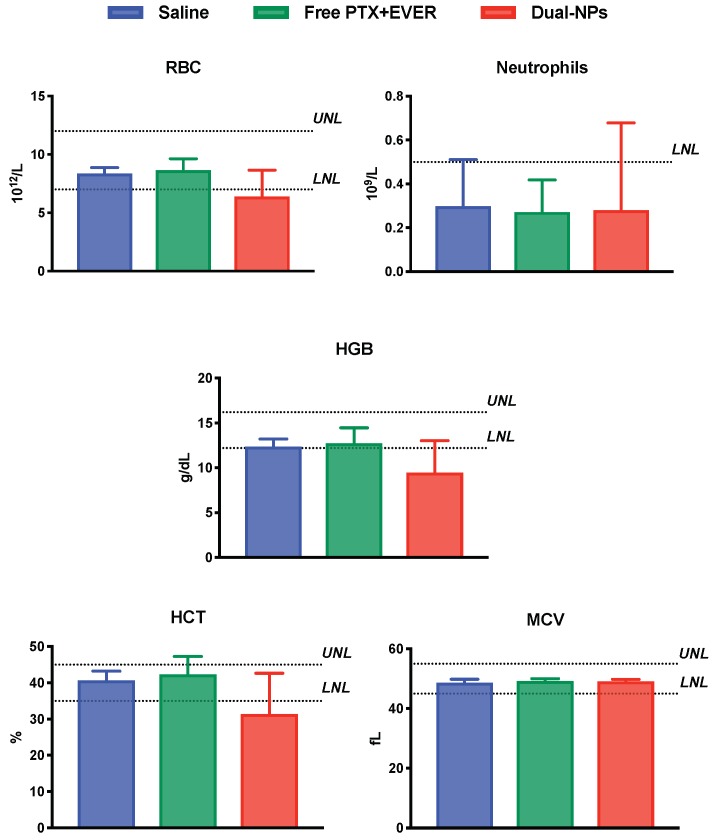
Red blood cell count (RBC), neutrophil count, hemoglobin concentration (HGB), hematocrit (HCT) and the mean corpuscular cell volume (MCV) in mice 2–3 days after receiving saline (blue), free paclitaxel and everolimus (PTX+EVER) (green), and dual-targeted PTX+EVER-loaded nanoparticles (Dual-NPs) (red) weekly for six weeks at a PTX-equivalent dose of 15 mg/kg. Data represent mean ± SD (*n* = 5–8).

For red blood cell count (RBC), hemoglobin concentration (HGB), hematocrit (HCT) and the mean corpuscular cell volume (MCV), values for all groups were within normal levels. Interestingly, the level of neutrophils in mice in all groups was comparable but below the lower normal limit. This outcome was previously seen by Nemzek and colleagues and was attributed to the strain of mice and sampling site used. It was found that in BALB/c mice, when blood is collected from the heart through cardiac puncture, neutrophil levels drop due to the reduced peripheral vascular resistance to the flow of blood cells [17,18]. Overall, there was no evidence of hemotoxicity in all groups (Figure 6).

**Figure 7:**
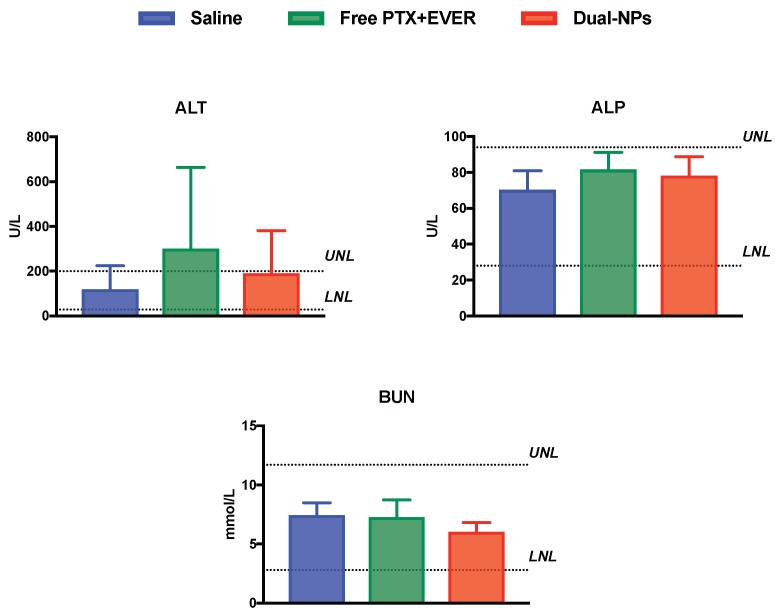
Alanine aminotransferase (ALT), alkaline phosphatase (ALP), and blood urea nitrogen (BUN) in mice 2–3 days after receiving saline (blue), free paclitaxel and everolimus (PTX+EVER) (green), and dual-targeted PTX+EVER-loaded nanoparticles (Dual-NPs) (red) weekly for 6 weeks at a PTX-equivalent dose of 15 mg/kg. Data represent mean ± SD (*n* = 5–8).

**Figure 8:**
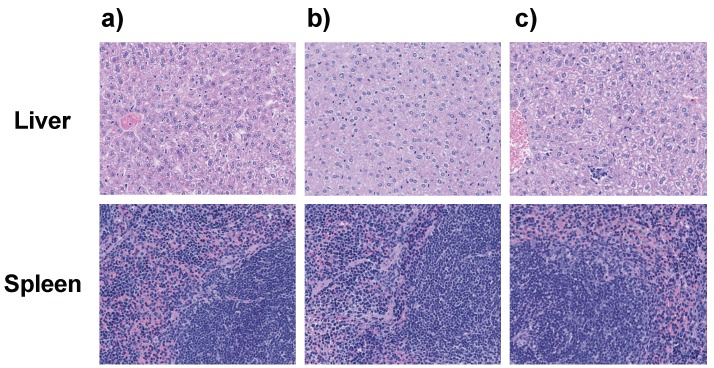
Cross-sections of the liver (upper) and spleen (lower) after staining with haematoxylin and eosin (H & E) 2–3 days after weekly injections for 6 weeks with saline (**a**), dual-targeted PTX+EVER-loaded nanoparticles (Dual-NPs) (**b**), and free paclitaxel and everolimus (PTX+EVER) (**c**) at a PTX-equivalent dose of 15 mg/kg. Magnification, 20X (*n* = 5–8).

Alkaline phosphatase (ALP) values (elevated in the blood most commonly by liver or bone disorders) and blood urea nitrogen (BUN) values (elevated in liver and kidney disorders) were within normal limits in all groups. Alanine aminotransferase (ALT) values (elevated with high specificity in liver disorders) were within normal limits in the saline and Dual-NPs groups, while mice in the free PTX+EVER group showed ALT levels higher than the upper limit. This increase in ALT levels, however, was not statistically significant relative to the saline and the Dual-NP groups (Figure 7). Therefore, there was no indication of liver toxicity in all groups. These results were further confirmed by the histopathological examination of formalin-embedded sections of the liver and spleen in all groups, which demonstrated normal histological appearance and no appreciable degeneration was observed (Figure 8).

To evaluate the kidney function, serum creatinine (SCR) levels were also quantified. SCR values in all groups were reported to be <18 µM/L. The low levels of SCR, along with the normal levels of blood urea nitrogen (BUN), negate the presence of kidney disorders—usually associated with high SCR levels—in all groups.

## 3. Discussion

Previously, the Dual-NPs demonstrated good stability in vitro with less than 50% PTX and EVER released after 72 h of incubation in BSA-supplemented media at 37 °C [11]. In the current study, PTX and EVER in Dual-NPs exhibited prolonged blood circulation whereas the free-drug combination showed rapid clearance. Consequently, the C_max_, AUCs, and half-lives of the drugs were significantly higher when administered in Dual-NPs in comparison to the free drug combination. In addition, the Dual-NPs maintained the synergistic molar ratio of PTX:EVER in the plasma for up to 24 h after administration. These results indicate that the Dual-NPs retained their stability in the blood circulation, which is essential for the successful co-delivery of PTX and EVER (at the optimal synergistic ratio) to the tumor site.

The stability of the Dual-NPs may be attributed mainly to their low drug to material ratio or drug loading level (i.e., 5.6% *w*/*w*). We have previously found that the drug to material ratio can significantly impact the stability of NPs. Dynamic light scattering (DLS) was used to measure the size of different untargeted NPs composed of mPEG_5000_-*b*-PLGA_15,800_ at different drug (i.e., PTX+EVER) to material ratios (6.5%, 8.9%, 11.7%, 15.3% and 16.1% *w*/*w*) to monitor their stability for 48 h (data not shown). Formulations with high drug to material ratios of 16.1% and 15.3%, significantly aggregated forming large clusters that could be seen with the naked eye within 6 h, indicating the low stability of the NPs. With drug to material ratios of 11.7% and 8.9%, aggregation of the NPs began after 24 h, leading to an increase in hydrodynamic diameter of about 40% and 70% of the initial size, respectively. On the other hand, with a drug to material ratio of 6.5%, the NPs maintained their initial size for 48 h at room temperature and this size was also maintained when the NPs were incubated in cell culture media containing 10% FBS at 37 °C. Therefore, the untargeted NP formulation with the lowest drug loading level (6.5%) was used for the development of Dual-NPs. The conjugation of the targeting moieties to the surface of the NPs resulted in a reduction in drug loading from 6.5% to 5.6% (*w*/*w*). Other studies have confirmed our findings and demonstrated that PLGA NPs with lower drug to material ratios are often associated with a higher degree of stability [19,20,21].

The development of a polymeric NP formulation that physically encapsulates a taxane (paclitaxel or docetaxel) and is stable in vivo has proven to be challenging [22,23,24]. Cynviloq (Genexol-PM) is the first block copolymer micelle (BCM) drug formulation approved for human use for treating different types of cancer including metastatic BC. Cynviloq is composed of the copolymer methoxy-PEG-*b*-poly(D,L-lactide) (mPEG-*b*-PDLLA) and has a drug loading level of 16% (*w*/*w*). In preclinical studies conducted in mice, the plasma AUC for PTX was 30% lower with Cynviloq than with Taxol despite the higher injected dose (50 mg/kg versus 20 mg/kg for Cynviloq and Taxol, respectively) [23]. The rapid release of PTX from the micelles after administration was attributed to the kinetic instability of this formulation [25].

Administration of PTX and EVER in the Dual-NPs resulted in only marginal improvements in tumor growth inhibition and survival in MDA-MB-231-H2N tumor-bearing mice relative to the free-drug combination. The Dual-NP formulation was compared to free PTX+EVER at chemically equivalent doses of both drugs. One may expect that a greater degree of antitumor efficacy could be achieved for the Dual-NP formulation if a higher dose had been administered. Given the good toxicity profile observed for this formulation, it is also likely that higher doses of the formulation could be safely administered. However, the low drug loading level of 5.6% (*w*/*w*) in the formulation limits the maximum dose of drug that can be administered (i.e., 15 mg/kg). Therefore, there must be a balance between high drug loading and sufficient in vivo stability in the design of the NP formulation to allow for superior antitumor effects.

Empty Dual-NPs are unlikely to have any toxic or therapeutic effects. PEG-*b*-PLGA is a biocompatible copolymer which has a long history of safe use in pre-clinical and clinical studies [26,27,28]. Fab fragments, unlike full antibodies, lack the fragment crystallizable region (Fc) and the growth-inhibitory properties of the full antibody responsible for the toxicities associated with the full antibody [29].

Importantly, as previously shown by other groups, active targeting of a NP formulation may not result in an increase in their tumor accumulation. In some cases, the accumulation of NPs at the tumor site, even with active targeting moieties at the surface of the NPs, can be mainly attributed to the enhanced permeability and retention (EPR) effect (passive targeting) [30,31]. There is great heterogeneity associated with the EPR effect. As a result, the accumulation of NPs can vary significantly in the same or different tumor types [32,33].

Peripheral neuropathy remains a persistent distressing side effect to patients receiving conventional PTX, interfering with their daily activities and negatively affecting their quality of life. Symptoms include numbness, tingling, burning, muscles weakness and cramping. This toxicity is seen in 70–90% of patients receiving conventional PTX, and even after discontinuation of therapy, it takes several months for symptoms of neuropathy to resolve [34,35]. With PTX becoming a key component of the standard treatment for BC in the adjuvant and metastatic setting, stopping or reducing drug administration due to neuropathy is undesirable as it can have significant implications on treatment outcomes [36].

Importantly, in the present study, neurotoxicity was found to be reduced with the administration of the drugs in the Dual-NPs relative to the free-drug combination. In addition, the formulation showed superior tolerability to free PTX+EVER in terms of body weight loss. This outcome could be attributed to the prolonged retention of the drugs within Dual-NPs in the blood circulation, enabling effective exploitation of the EPR effect which leads to selective tumor accumulation and, subsequently, reduced systemic drug exposure and associated toxicities [37]. In addition, the use of polymeric NPs composed of the biocompatible polymer PEG-*b*-PLGA, which has a good safety profile allowed for the elimination of the small-molecule surfactant Cremophor EL, which has been associated with PTX-induced neurotoxicity [1].

## 4. Methods

### 4.1. Tumor Cells

The human BC cell line, MDA-MB-231-H2N, was provided by Dr. Raymond Reilly (University of Toronto, ON). The cells were cultured in Dulbecco’s Modified Eagle Medium/Nutrient Mixture F-12) (DMEM/F12) with 10% fetal bovine serum (FBS) at 37 °C in 5% CO_2_ and 90% relative humidity. The medium was supplemented with penicillin/streptomycin solution to 1% of final volume. Based on previous assessments of HER2 and EGFR expression by radioligand binding assays, MDA-MB-231-H2N has moderate HER2 (4.5 × 10^5^ receptors/cell) and EGFR expression (4.8 × 10^5^ receptors/cell) [38]. Based on these receptor densities, the cell line was designated as HER2_mod_/EGFR_mod_.

### 4.2. Animals and Tumor Model

Four-week old, healthy, female BALB/c mice were purchased from Charles River Laboratories (Wilmington, MA, USA) for the pharmacokinetics and toxicity studies. Four-week old, female NOD/SCID mice were purchased from an in-house breeding facility for the biodistribution studies and from Charles River Laboratories for the efficacy studies. Animal studies were conducted following protocols approved by University of Toronto’s Animal Care Committee (approval No. 20012011, 7 August 2017).

### 4.3. Preparation of Dual-NPs

PTX+EVER-loaded NPs were prepared as previously described [6]. Briefly, PTX, EVER, mPEG_5000_-*b*-PLGA_15,800_ and COOH-PEG-*b*-PLGA (at ~1% of total polymer weight) were dissolved in acetone. Under magnetic stirring, following complete dissolution, the organic phase was added drop-wise to deionized water. The mixture was left under vacuum overnight at RT to evaporate the organic phase. The formulation was filtered and purified by ultrafiltration (3500 g for 10 min at RT) using ultracentrifugal filter units (Nominal molecular weight limit (NMWL) of 100 kDa) (Millipore Canada Ltd., Etobicoke, Canada). The HER2-targeted trastuzumab (TmAb) and EGFR-targeted panitumumab (PmAb) Fab fragments were generated by digestion of TmAb and PmAb IgG using immobilized papain following a well-established method [39]. The purity and identity of the Fab fragments were confirmed by sodium dodecyl sulfate polyacrylamide gel electrophoresis (SDS-PAGE) and size exclusion high performance liquid chromatography (SEC-HPLC). The Fab fragments were conjugated to previously prepared PTX+EVER-loaded NPs using the amine coupling method similar to that described by Gao et al. [40]. Briefly, the NP suspension was incubated with N-Hydroxysuccinimide (NHS) (100 mM) and N-Ethyl-N′-(3-dimethylaminopropyl)carbodiimide (EDC) (400 mM) (Sigma-Aldrich, Etobicoke, Canada) for 15 min at RT. The resulting NHS-activated NPs were incubated with TmAb Fab fragments to prepare T-NPs, or a 1:1 mix of TmAb and PmAb Fab fragments (5 mg/mL in PBS, pH 7) for 1 h at RT. The resulting suspension of the dual-targeted PTX+EVER-loaded NPs (Dual-NPs) was purified by ultrafiltration (3500 g for 10 min at RT) using ultracentrifugal filter units (NMWL of 100 kDa). The purification of Dual-NPs was repeated three times. The amount of Fab fragment conjugated to the NPs’ surface, was quantified using the Bradford assay, and found to be approximately 13 μg Fab per 1 mg of NPs (resulting in a ligand density of ca. 12 ligands per NP).

### 4.4. Pharmacokinetic Study

Healthy BALB/c female mice were injected intravenously (i.v.) via the tail vein with either the free PTX+EVER combination or the Dual-NP formulation at a PTX-equivalent dose of 15 mg/kg and EVER-equivalent dose of 7.5 mg/kg (i.e., PTX to EVER molar ratio of 1:0.5). The free-drug combination was dissolved in a 1:1 mixture of Cremophor EL and anhydrous ethanol. The resulting yellow viscous mixture was then diluted with saline and vortexed for 20 s to obtain a clear solution of the drugs. Mice were sacrificed by cardiac puncture under anesthesia at selected time points post-administration. Blood was collected using heparinized tubes and centrifuged at 15,000 rpm for 10 min at 4 °C to isolate plasma. Plasma was stored at −80 °C prior to analysis. Acetonitrile was added to the plasma at a 2:1 (*v*/*v*) ratio and the mixture was centrifuged for 10 min at 5000 g at 4 °C to precipitate the protein content of plasma. Supernatant was collected and the drugs were extracted by liquid-liquid extraction using t-butylmethylether (TBME). The organic phase was collected and evaporated under nitrogen before resuspending in the mobile phase (acetonitrile and water, 1:1 *v*/*v*).

PTX and EVER were quantified using HPLC as previously reported [6]. In brief, PTX and EVER were measured by isocratic reverse-phase HPLC analysis (Agilent) using a C18 column. Acetonitrile and water (50:50 *v*/*v*) or methanol, water, trifluoroacetic acid (95:4.9:0.1 *v*/*v*) were used as mobile phases for PTX and EVER, respectively, at a flow rate of 1.0 mL/min. A diode array detector was set at wavelengths of 227 nm and 279 nm for detection of PTX and EVER, respectively (Agilent, CA, USA). The extraction efficiencies were 86% and 81%, the limit of detection (LOD) was 0.1 µg/mL and 0.05 µg/mL (at a signal to noise ratio of ≥3), and the limit of quantification (LOQ) was 0.4 µg/mL and 0.1 µg/mL for PTX and EVER, respectively.

Non-compartmental analysis was used to calculate all pharmacokinetic parameters as previously reported [41]. The half-life at the terminal phase (t_1/2_z) was calculated by dividing 0.693 by the slope of the terminal phase (k). The AUC_0–t_ was calculated using the linear trapezoidal rule where t is the time of the last measurable concentration. AUC_t–inf_ was calculated by dividing the last detected concentration by the slope of the terminal phase (k). The clearance (Cl) was estimated by the equation Cl = dose/AUC_0-inf_, and the volume of distribution (V_d_) by V_d_ = dose/(AUC_0-t_ K).

### 4.5. Tumor Inoculation

MDA-MB-231-H2N tumor-bearing female NOD/SCID mice were used for the biodistribution and efficacy studies. 1.0 × 10^6^ MDA-MB-231-H2N cells suspended in a 100 μL mixture of culture medium and Matrigel (1:1 *v*/*v*) were injected subcutaneously in the right hind limb of mice. When the tumor size reached 5–8 mm in diameter, mice were randomly allocated to the treatment or control groups. 

### 4.6. Biodistribution Study

NOD/SCID female mice bearing MDA-MB-231-H2N tumors were injected i.v. into the tail vein with either free PTX+EVER or Dual-NPs at a PTX-equivalent dose of 15 mg/kg and EVER-equivalent dose of 7.5 mg/kg (PTX:EVER at a molar ratio of 1:0.5). At selected time points post-administration, mice were sacrificed and tumors, spleens, livers, hearts and kidneys were collected and stored at −80 °C. Organs obtained were homogenised in 0.9% NaCl and the resulting homogenate was extracted and quantified using the methods described above for plasma. The extraction efficiencies were 80% and 76% for PTX and EVER, respectively. The LOD was 0.2 µg/mL (at a signal to noise ratio of ≥3) for both drugs, and the LOQ was 0.5 µg/mL and 0.4 µg/mL for PTX and EVER, respectively. Data were analyzed using the Student’s *t* test, and *p*-values < 0.05 were considered statistically significant.

### 4.7. Efficacy Studies

NOD/SCID female mice bearing MDA-MB-231-H2N tumors received either saline, free PTX+EVER, or Dual-NPs as an i.v. dose administered weekly for eight consecutive weeks at a PTX-equivalent dose of 15 mg/kg and EVER-equivalent dose of 7.5 mg/kg (PTX: EVER at a molar ratio of 1:0.5). Tumor growth was monitored by caliper measurements of tumor width (*w*) and length (*l*). Tumor volume was determined using the formula v  =  π/6*l*(*w*^2^), while drug toxicity was determined by monitoring changes in animal behavior and body weight. Groups were excluded from the tumor volume efficacy study when the first mouse in each group reached the ethical endpoint. Statistical significance was calculated by the two-way ANOVA (analysis of variance) test.

Mice were sacrificed and removed from the survival study when they reached the ethical endpoint—if any of the following was observed: hunched or abnormal posture; weight loss exceeding 20% of normal body weight; tumor mass compromising normal behavior, ambulation, limited food and water intake; failure to groom (piloerection). Survival data were plotted using Kaplan-Meier survival analysis, and statistical significance was determined using the log-rank test. 

### 4.8. Toxicity Studies

Four-week old, healthy, female BALB/c mice were given the following weekly treatments via i.v. tail vein injection: Dual-NPs, free PTX+EVER at a PTX-equivalent dose of 8 mg/kg (low-dose study) or 15 mg/kg (high-dose study) (PTX:EVER at a molar ratio of 1:0.5). The control group received weekly injections of saline for six consecutive weeks. Mice were sacrificed two to three days after receiving the last injection and the severity of neurotoxicity was examined using light microscopy. Segments of the sciatic nerve were embedded in formalin. Sections of 4 µm in thickness were stained with haematoxylin and eosin (H & E) before examination under light microscopy by a veterinary pathologist blinded to the groups to determine the degenerative changes in the myelinated nerve fibers. 

For mice receiving the higher dose of the drugs, blood was collected into heparinized tubes for biochemistry analysis and in Ethylenediaminetetraacetic acid (EDTA) tubes for complete blood count analysis (CBC). Blood biochemical markers were measured using the VetScan VS2 (Abaxis, Union City, CA, USA) Comprehensive Diagnostic Profile, while CBC analysis was conducted using the VetScan HM5 v2.2 hematology analyzer. Mice in this group were also evaluated for liver and spleen toxicity by histopathological examination of formalin-embedded sections of the liver and spleen. Data were analyzed using the Student’s t test, and *p*-values < 0.05 were considered statistically significant.

## 5. Conclusions

While maintaining therapeutic efficacy, Dual-NPs were successful in improving the toxicity profile of PTX relative to the free-drug combination. In addition to showing preliminary evidence of reducing neurotoxicity, Dual-NPs were significantly more tolerable than the free-drug combination with regards to body weight loss when administered at the same dose. Considering the severe toxicities associated with conventional PTX (Taxol), especially at this time when the overall survival rates for BC have improved; the development of well-tolerated medicines should be prioritized alongside the search for curative measures.

## Figures and Tables

**Table 1:** Drug loading, size, and surface charge of dual-targeted paclitaxel (PTX)+everolimus (EVER)-loaded nanoparticles (Dual-NPs).

Formulation	PTX:EVER LC (wt%) ^a^	PTX (mg/mL)	EVER (mg/mL)	PTX:EVER Molar Ratio	Size (nm)	Zeta Potential (mV)
Dual-NPs	5.6 ± 1.1	1.2 ± 0.2	0.5 ± 0.1	1:0.37	101.3 ± 6.3	−11.3 ± 2.1

^a^ LC% = Loading content.

**Table 2:** Pharmacokinetics parameters for the plasma concentrations of paclitaxel (PTX) and everolimus (EVER) after a single intravenous administration of dual-targeted PTX+EVER-loaded nanoparticles (Dual-NPs) versus free PTX+EVER combination to healthy BALB/c female mice.

Treatment		C_max_ (µg/mL)	T_1/2_z (h)	AUC_0–t_ (µg h mL^−1^)	AUC_0–inf._ (µg h mL^−1^)	CL_tot_ (mL h kg^−1^)	V_d_ (mL kg^−1^)
Free Combination	PTX	64.91	1.88	170.49 ^a^	172.09	81.35	222.52
	EVER	38.41	1.02	83.6 ^b^	84.88	88.35	131.73
Dual-NPs	PTX	458.03	5.17	3666.65 ^c^	3671.59	3.81	28.47
	EVER	351.25	3.78	1792.52 ^d^	1814.83	4.13	22.85

C_max_ = the maximum plasma concentration; t_1/2_z = half-life at the terminal phase; AUC = area under the curve; CL_tot_ = total body clearance; V_d_ = volume of distribution. ^a^ AUC_0–12 h_, ^b^ AUC_0–6 h_, ^c^ AUC_0–48 h_, ^d^ AUC_0–24 h_. (*n* = 5).

## References

[1] Gelderblom H., Verweij J., Nooter K., Sparreboom A. (2001). Cremophor EL: The drawbacks and advantages of vehicle selection for drug formulation. Eur. J. Cancer.

[2] Hidalgoa M., Sánchez-Morenob C., Pascual-Teresa S. (2010). Flavonoid–flavonoid interaction and its effect on their antioxidant activity. Food Chem..

[3] Chou T., Talalay P. (1984). Quantitative Dose-effect relationships: The combined effects of multiple drug or enzyme inhibitors. Adv. Enzym. Regul..

[4] Mignani S., Bryszewska M., Klajnert-Maculewicz B., Zablocka M., Majoral J.P. (2015). Advances in combination therapies based on nanoparticles for efficacious cancer treatment: An analytical report. Biomacromolecules.

[5] Jadia R., Scandore C., Rai P. (2016). Nanoparticles for Effective Combination Therapy of Cancer. Int. J. Nanotechnol. Nanomed..

[6] Houdaihed L., Evans J.C., Allen C. (2018). Codelivery of Paclitaxel and Everolimus at the Optimal Synergistic Ratio: A Promising Solution for the Treatment of Breast Cancer. Mol. Pharm..

[7] Gonzalez-Angulo A.M., Akcakanat A., Liu S., Green M.C., Murray J.L., Chen H., Palla S.L., Koenig K.B., Brewster A.M., Valero V. (2014). Open-label randomized clinical trial of standard neoadjuvant chemotherapy with paclitaxel followed by FEC versus the combination of paclitaxel and everolimus followed by FEC in women with triple receptor-negative breast cancer. Ann. Oncol..

[8] Campone M., Levy V., Bourbouloux E., Berton Rigaud D., Bootle D., Dutreix C., Zoellner U., Shand N., Calvo F., Raymond E. (2009). Safety and pharmacokinetics of paclitaxel and the oral mTOR inhibitor everolimus in advanced solid tumours. Br. J. Cancer.

[9] Approved Drugs—FDA Approves Liposome-Encapsulated Combination of Daunorubicin-Cytarabine for Adults with Some Types of Poor Prognosis AML. https://www.fda.gov/Drugs/InformationOnDrugs/ApprovedDrugs/ucm569950.htm.

[10] Lancet J.E., Uy G.L., Cortes J.E., Newell L.F., Lin T.L., Ritchie E.K., Stuart R.K., Strickland S.A., Hogge D., Solomon S.R. (2016). Final results of a phase III randomized trial of CPX-351 versus 7+3 in older patients with newly diagnosed high risk (secondary) AML. J. Clin. Oncol..

[11] Houdaihed L., Evans J., Allen C. (2019). Dual-Targeted Delivery of Nanoparticles Encapsulating Paclitaxel and Everolimus: A Novel Strategy to Overcome Breast Cancer Receptor Heterogeneity.

[12] Van der Meel R., Vehmeijer L.J.C., Kok R.J., Storm G., van Gaal E.V.B. (2013). Ligand-targeted particulate nanomedicines undergoing clinical evaluation: Current status. Adv. Drug Deliv. Rev..

[13] Lux M., Nabieva N., Hartkopf A.D., Huober J., Volz B., Taran F.-A., Overkamp F., Kolberg H.-C., Hadji P., Tesch H. (2019). Therapy Landscape in Patients with Metastatic HER2-Positive Breast Cancer: Data from the PRAEGNANT Real-World Breast Cancer Registry. Cancers.

[14] DiGiovanna M.P., Stern D.F., Edgerton S.M., Whalen S.G., Moore D., Thor A.D. (2005). Relationship of epidermal growth factor receptor expression to ErbB-2 signaling activity and prognosis in breast cancer patients. J. Clin. Oncol..

[15] Owens D.E., Peppas N.A. (2006). Opsonization, biodistribution, and pharmacokinetics of polymeric nanoparticles. Int. J. Pharm..

[16] Hamaguchi T., Matsumura Y., Suzuki M., Shimizu K., Goda R., Nakamura I., Nakatomi I., Yokoyama M., Kataoka K., Kakizoe T. (2005). NK105, a paclitaxel-incorporating micellar nanoparticle formulation, can extend in vivo antitumour activity and reduce the neurotoxicity of paclitaxel. Br. J. Cancer.

[17] Remick D., Nemzek J.A., Bolgos G.L., Williams B.A., Remick D.G. (2001). Differences in normal values for murine white blood cell counts and other hematological parameters based on sampling site. Inflamm. Res..

[18] Quimby F.H., Goff L.G. (1952). Effect of Source of Blood Sample on Total White Cell Count of the Rat. Am. J. Physiol..

[19] Musacchio T., Laquintana V., Latrofa A., Trapani G., Torchilin V. (2009). PEG-PE micelles loaded with paclitaxel and surface-modified by a PBR-ligand: Synergistic anticancer effect. Mol. Pharm..

[20] Abouzeid A.H., Patel N.R., Torchilin V.P. (2014). Polyethylene glycol-phosphatidylethanolamine (PEG-PE)/vitamin e micelles for co-delivery of paclitaxel and curcumin to overcome multi-drug resistance in ovarian cancer. Int. J. Pharm..

[21] Averineni R., Shavi G.V., Gurram A.K., Deshpande P.B., Arumugam K., Maliyakkal N., Meka S.R., Nayanabhirama U. (2010). PLGA 50:50 nanoparticles of paclitaxel: Development, in vitro anti-tumor activity in BT-549 cells and in vivo evaluation. Bull. Mater. Sci..

[22] Hrkach J., Von Hoff D., Mukkaram Ali M., Andrianova E., Auer J., Campbell T., De Witt D., Figa M., Figueiredo M., Horhota A. (2012). Preclinical Development and Clinical Translation of a PSMA-Targeted Docetaxel Nanoparticle with a Differentiated Pharmacological Profile. Sci. Transl. Med..

[23] Kim S.C., Kima D.W., Shima Y.H., Banga J.S., Oh H.S., Kim S.W., Seoa M.H. (2001). In vivo evaluation of polymeric micellar paclitaxel formulation: Toxicity and efficacy. J. Control Release.

[24] Chen H., Kim S.W., He W., Wang H.F., Low P.S., Park K., Cheng J.X. (2008). Fast release of lipophilic agents from circulating PEG-PDLLA micelles revealed by in vivo Forster resonance energy transfer imaging. Langmuir.

[25] Kim T.Y., Kim D.W., Chung J.Y., Shin S.G., Kim S.C., Heo D.S., Kim N.K., Bang Y.J. (2004). Phase I and pharmacokinetic study of Genexol-PM, a Cremophor-free, polymeric micelle-formulated paclitaxel, in patients with advanced malignancies. Clin. Cancer Res..

[26] Beletsi A., Panagi Z., Avgoustakis K. (2005). Biodistribution properties of nanoparticles based on mixtures of PLGA with PLGA-PEG diblock copolymers. Int. J. Pharm..

[27] Koopaei M.N., Khoshayand M.R., Mostafavi S.H., Amini M., Khorramizadeh M.R., Tehrani M.J., Atyabi F., Dinarvanda R. (2014). Docetaxel Loaded PEG-PLGA Nanoparticles: Optimized Drug Loading, In-vitro Cytotoxicity and In-vivo Antitumor Effect. Iran. J. Pharm. Res..

[28] Rezvantalab S., Drude N.I., Moraveji M.K., Güvener N., Koons E.K., Shi Y., Lammers T., Kiessling F. (2018). PLGA-based nanoparticles in cancer treatment. Front. Pharmacol..

[29] Neve R.M., Nielsen U.B., Kirpotin D.B., Poul M., Marks J.D., Benz C.C. (2001). Biological Effects of Anti-ErbB2 Single Chain Antibodies Selected for Internalizing Function. Biochem. Biophys. Res. Commun..

[30] Kirpotin D.B., Drummond D.C., Shao Y., Shalaby M.R., Hong K., Nielsen U.B., Marks J.D., Benz C.C., Park J.W. (2006). Antibody targeting of long-circulating lipidic nanoparticles does not increase tumor localization but does increase internalization in animal models. Cancer Res..

[31] Carstens M.G., de Jonga P., van Nostrum C.F., Kemmink J., Verrijk R., de Leede L., Crommelin D., Hennink W.E. (2008). The effect of core composition in biodegradable oligomeric micelles as taxane formulations. Eur. J. Pharm. Biopharm..

[32] Harrington K.J., Mohammadtaghi S., Uster P.S., Glass D., Peters A.M., Vile R.G., Stewart J.S. (2001). Effective Targeting of Solid Tumors in Patients With Locally Advanced Cancers by Radiolabeled Pegylated Liposomes Effective Targeting of Solid Tumors in Patients With Locally Advanced Cancers by Radiolabeled Pegylated Liposomes. Clin. Cancer Res..

[33] Maeda H. (2015). Toward a full understanding of the EPR effect in primary and metastatic tumors as well as issues related to its heterogeneity. Adv. Drug Deliv. Rev..

[34] Starobova H., Vetter I., Vetter I. (2017). Pathophysiology of Chemotherapy-Induced Peripheral Neuropathy. Front. Mol. Neurosci..

[35] Scripture C.D., Figg W.D., Sparreboom A. (2006). Peripheral Neuropathy Induced by Paclitaxel: Recent Insights and Future Perspectives. Curr. Neuropharmacol..

[36] Waseem A., Rao R.R., Agarwal A., Saha R., Bajpai P., Qureshi S., Mittal A. (2018). Incidence of Neuropathy with Weekly Paclitaxel and Role of Oral Glutamine Supplementation for Prevention of Paclitaxel Induced Peripheral Neuropathy Randomized Controlled Trial. Indian J. Med. Paediatr. Oncol..

[37] Houdaihed L., Evans J.C., Allen C. (2017). Overcoming the Road Blocks: Advancement of Block Copolymer Micelles for Cancer Therapy in the Clinic. Mol. Pharm..

[38] Razumienko E., Dryden L., Scollard D., Reilly R.M. (2013). MicroSPECT/CT Imaging of Co-Expressed HER2 and EGFR on Subcutaneous Human Tumor Xenografts in Athymic Mice Using 111In-Labeled Bispecific Radioimmunoconjugates. Breast Cancer Res. Treat..

[39] Hoang B., Reilly R.M., Allen C. (2012). Block Copolymer Micelles Target Auger Electron Radiotherapy to the Nucleus of HER2-Positive Breast Cancer Cells. Biomacromolecules.

[40] Gao J., Kou G., Wang H., Chen H., Li B., Lu Y., Zhang D., Wang S., Hou S., Qian W. (2009). PE38KDEL-loaded anti-HER2 nanoparticles inhibit breast tumor progression with reduced toxicity and immunogenicity. Breast Cancer Res. Treat..

[41] Sparreboom A., van Tellingen O., Nooijen W.J., Beijnen J.H. (1996). Nonlinear Pharmacokinetics of Paclitaxel in Mice Results from the Pharmaceutical Vehicle Cremophor EL. Cancer Res..

